# Changing Stem Cell Dynamics during Papillomavirus Infection: Potential Roles for Cellular Plasticity in the Viral Lifecycle and Disease

**DOI:** 10.3390/v9080221

**Published:** 2017-08-12

**Authors:** Katerina Strati

**Affiliations:** Department of Biological Sciences, University of Cyprus, 1 University Avenue, Nicosia, 2109, Cyprus; strati@ucy.ac.cy; Tel.: +357 22 892884

**Keywords:** HPV, stem cells, cellular plasticity

## Abstract

Stem cells and cellular plasticity are likely important components of tissue response to infection. There is emerging evidence that stem cells harbor receptors for common pathogen motifs and that they are receptive to local inflammatory signals in ways suggesting that they are critical responders that determine the balance between health and disease. In the field of papillomaviruses stem cells have been speculated to play roles during the viral life cycle, particularly during maintenance, and virus-promoted carcinogenesis but little has been conclusively determined. I summarize here evidence that gives clues to the potential role of stem cells and cellular plasticity in the lifecycle papillomavirus and linked carcinogenesis. I also discuss outstanding questions which need to be resolved.

## 1. Introduction

Human papillomavirus (HPV) infection occurs via micro wounds which allow the virus access to the basal layer of stratified epithelia. This target site of infection has been the subject of intense scrutiny, leading to significant progress regarding the molecular mechanisms governing the attachment and eventual entry of the virus into initially infected cells [1,2]. However, the basal layer of stratified epithelia has a relatively heterogeneous composition containing committed undifferentiated cells, progenitors, and also the tissue stem cells [3]. It is not understood whether these are subject to differential infection and whether the fate of infected cells varies based on cell identity prior to infection. Nevertheless, it has been proposed that the virus may be more successfully maintained [4,5], and indeed infected cells are more likely to proceed to tumorigenesis when the initially infected cell is a tissue stem cell [6,7]. To this day there is little evidence to conclusively resolve this question. Yet as progress is being made to better understand the role of “stemness” in carcinogenesis overall, the question of its role in HPV-mediated carcinogenesis and potentially the viral lifecycle, grows more intriguing. Furthermore, our evolving understanding of cellular plasticity raises new possibilities which may be at play during the HPV lifecycle and pathogenesis. In this review, I will discuss the available evidence and its implications and propose significant questions which remain to be addressed.

## 2. The Target Cell of Papillomavirus Infection

Papillomaviruses can productively infect both mucosal and cutaneous stratified epithelia. Infection and the ensuing life cycle of the virus are intimately linked to the differentiation program of the tissue [8,9] thus our reconstruction of these events relies on the solid understanding of tissue biology. The skin is an easily accessible model tissue for stratified epithelial differentiation, homeostasis, and regeneration due to its rapid turnover and ability to regenerate quickly upon mechanical injury. Much of our knowledge pertaining to the role of stem cell populations in these processes derives from studying the cutaneous epithelia in mouse models [3]. The characteristics of cutaneous epithelia and their stem cell populations may or may not extrapolate to the mucosal tissues. However, since the cervical stem cells have not been conclusively characterized the comparison of the cervical and cutaneous epithelia is useful and necessary. In stratified epithelia, the stem cells orchestrate tissue homeostasis as well as acute regeneration [3]. From work done in mouse models we know that different stem cell pools are primarily responsible for replacing lost cells during homeostasis and regeneration [10,11]. Genetic ablation experiments have also provided evidence that a certain amount of plasticity exists (for example if bulge stem cells are ablated, neighboring cells can replenish the stem cell niche) [11,12]. Stem cells in cutaneous epithelia are typically slow-cycling and can perform asymmetric division, dependent on the cellular niche, generating one stem and one transient amplifying/progenitor cell. Of course, during wound repair the balance of asymmetric division may be shifted to replenish stem cell populations to homeostatic levels [11]. Progenitors can then undergo large numbers of cell divisions and gradual differentiation helping to replenish the tissue.

Studies using HPV virus like particles (VLPs), or infectious virions for attachment and viral entry argue against the exclusive targeting of a small subpopulation such as the tissue stem cells. Rather, the virus associates with the exposed basement membrane at a site of wounding, and furin cleavage leads to conformational changes on the capsid which allow it to attach to nearby basal cells via a receptor which recognizes L1 capsid protein [1,2]. The high prevalence of infection with HPVs [13] seen in populations worldwide also provides an argument against a model in which the virus exclusively targets a rare subpopulation. It is likely that both stem and non-stem cells adjacent to a site of wounding can be infected. If there are smaller differences of susceptibilities to infection between cell types, those would be technically difficult to quantify at least in vivo. However, it is conceivable that the outcome of infection differs depending on the cell type infected.

## 3. A Cell Reservoir for Long-Term Viral Maintenance

If one takes for granted the more likely scenario that both stem and committed cells can be the targets of infection, then a vital question concerns the fate of infection in a stem as opposed to a committed cell. A popular hypothesis has been that long-term maintenance of the viral DNA can take place within infected tissue stem cells [4,5]. This is particularly relevant to low-level, asymptomatic, persistent infection which has been argued to be the source of future reactivation. [14,15]. During the long period between initial infection and disease development the viral DNA may be able to persist in a small subset of cells and is clinically undetectable. The reasons for lack of detection are likely linked to the small number of cells harboring genomes and low levels of viral replication. Such infections can escape immunological control later in life and lead to disease. Reactivation is marked by higher levels of viral replication and transcription which facilitate clinical detection. While reactivation has not been conclusively determined to occur in humans it is strongly supported by evidence obtained using rabbit papillomaviruses as a model [5,16]. In a cottontail rabbit papillomavirus (CRPV) infection model, viral genomes were detected in an area of the hair follicle which coincides with cells with in vitro clonogenic activity [16]. In a rabbit oral papillomavirus (ROPV) infection model it has been shown that the virus DNA can persist for long periods of time in a subset of the basal epithelial cells [5]. The authors speculate that these cells harboring viral DNA represent the epithelial stem cells. This scenario is not unlikely when one considers the contrast between the timeline of disease reactivation (over a year following infection) compared to the quick regenerative pace of the epithelium. One would predict that upon infection of a committed cell, the progeny cells harboring the virus would be cleared from the tissue in a matter of days/months. Thus, it is reasonable to hypothesize that cells which are maintained and harbor genomes for long periods of time represent a type of tissue stem cell. However, little is known about the molecular characteristics of a stem cell in the rabbit oral epithelium. Much less can be inferred about the identity of the cell prior to infection, thus it is difficult to assess this claim conclusively.

Hair follicle stem cells have also been proposed as the reservoirs for human cutaneous HPV infections. DNA of β-papillomaviruses is frequently isolated from plucked eyebrow hairs [17,18] suggesting that a cell type at this location is the long-term reservoir of the virus. β-Papillomaviruses are emerging as potential co-factors in a subset of non-melanoma skin cancers [19,20]. Definitively pinpointing the exact nature of the cell reservoir may be important for predicting the possibility of viral reactivation in at-risk populations (*Epidermodysplasia verruciformis* (EV) patients, transplant recipients etc.) [20].

The inability to define the concrete characteristics of the human cervical stem cells has complicated studies aiming to understand the maintenance of the human mucosotropic viruses. It is thought that the transformation zone which is characterized by a transition from columnar to squamous epithelium is the site of the so-called reserve cells which may act as the cervical stem cells. Some markers have been proposed for reserve cells (e.g., Keratin 17 (K17), p63, Keratin 7 (K7), etc.) [21,22] but the dearth of healthy human biopsy material and the loose anatomic equivalence of the mouse cervix have hindered functional studies of stemness on such putative stem cell populations. Importantly, a subset of cells in the transformation zone have been shown to be susceptible to HPV infection, and high-grade lesions stemming from this area are more likely to progress to carcinoma in situ [22]. Furthermore, lesions share the expression of markers of this area—e.g., K7, matrix metalloproteinase-7 (MMP-7), cluster of differentiation 63 (CD63)—and this immunophenotype was not regenerated after removal, in other sites, or by HPV oncogene expression in keratinocytes. It is likely that these junctional cells represent the source of at least some cervical malignancies and may represent a cervical stem cell population. Reserve cells can likely serve as a site for infection and potentially a viral reservoir. There are however HPV lesions which can be detected in other mucosal sites (e.g., the vagina) which do not share this anatomic feature thus it is unlikely that these cells are the unique targets of infection, maintenance or transformation.

## 4. Changes in Tissue Stem Cell Dynamics during Infection

Infected tissue stem cells are of interest due to their potential links to carcinogenesis. However, more recently stem cells have also been implicated in the tissue response to infection. There is an emerging understanding that tissue stem cells have evolved to respond directly both to commensal and pathogenic microbes as evidenced by the expression of pattern recognition receptors (PRRs) in tissue stem cells [23,24,25]. In addition to inflammatory signals (discussed in a later section of this review), tissue stem cells have been shown to respond to the presence of microbes in ways which define the balance between maintaining tissue health or disease development. The paradigm has been set by studies in the gut where expression of nucleotide-binding oligomerization domain-containing protein 2 (Nod2) [24] and Toll-like receptor 4 (TLR4) [25] receptors in intestinal stem cells has provided a direct link for the interaction of the stem cells with tissue commensals via the recognition of peptidoglycan and lipopolysaccharide (LPS), respectively. This interaction has been shown to be critical to tissue regeneration and homeostasis suggesting a direct link between microbes and tissue stem cells as essential to tissue health. Of course, tissue stem cell dynamics have also been shown to be perturbed by pathogenic bacteria in the gut [26,27] and other tissues such the urogenital tract where pathogenic *Escherichia coli* [28] mobilize tissue stem cells and progenitors during pathogenesis.

While the effects of infection on tissue stem cell dynamics are less well understood in cutaneous and mucosal epithelia compared to the gut, studies investigating the expression of viral gene products on skin stem cell populations suggest that important changes occur. Compelling evidence regarding the changes in stem cell dynamics during papillomavirus infection comes from studies using transgenic animals for both mucosotropic [6,29] and cutaneous HPVs [30]. The available evidence for HPV16 converges towards a model where the expression of early gene products pushes the tissue stem cells towards a hybrid state: one which retains typical markers of stem cells (e.g., K15) [6,29], but also expresses atypical markers (e.g., P-cadherin) [6] and loses key functional characteristics such as quiescence. Loss of quiescence and increased mobilization of the stem cells has been reported both upon individual expression of HPV16 E6 and HPV16 E7 likely through different pathways [29]. This change in stem cell dynamics may represent a critical aspect in the process of viral carcinogenesis. Stem cell quiescence is a tumor refractory state and its absence may render the tissue more vulnerable to additional carcinogenic insult [31]. Interestingly, HPV-associated tumorigenesis has also been linked to non-quiescent, skin stem cell populations [32] likely to be hierarchically linked to quiescent populations [33]. One study showed that in mice, tumors induced by HPV16 oncogenes are derived from descendants of leucine-rich repeat-containing G-protein coupled receptor 5 (LGR5)-positive stem cells [6]. These are long-lived, non-quiescent cells in the hair follicle, which have been shown to actively contribute to hair-follicle growth. Combined, these findings may represent a common pathway in which HPV infection can lead to carcinogenesis by increasing stem cell mobilization and promoting a stem cell state which is receptive to further oncogenic changes. These studies were performed in mice, using transgenic animals which express the viral oncogenes throughout the basal layer of the epithelium. Thus, it is difficult to precisely extrapolate these findings to what happens during human infection. But the results support the notion that expression of viral oncogenes in cells with stem cell properties has the potential to profoundly alter the behavior of these cells, their susceptibility to carcinogenic insult, and rendering them more likely to contribute to carcinogenesis.

## 5. Changes in Cellular Plasticity during Infection

Initial focus has been on uncovering how the behavior of stem cells may change upon papillomavirus infection. There is however also evidence which supports an alternate, not mutually exclusive scenario: that during infection it is possible that reprogramming events can contribute to the emergence of stem-cell like cells.

The increased stem cell mobilization seen in transgenic animals is also concurrent with the expression of stem cell markers such as Keratin15 (K15) outside the typical stem cell niche [6,29]. The expression of early genes from the cutaneous HPV8 has also been shown to lead to an expansion of stem cell markers, specifically leucine-rich repeats and immunoglobulin-like domains 1 (Lrig1) [30] in a model expressing the early genes of HPV8 in the skin epithelium of mice. Interestingly similar Lrig1 expression pattern was also seen in biopsies from EV patients. This may represent a field cancerization effect critical to β-papillomavirus-induced carcinogenesis which does not implicate the integration of the virus in developing carcinomas as seen in alpha-papillomaviruses.

In light of technologies describing cellular reprogramming developed after Yamanaka’s seminal discoveries in 2006 [34], the re-expression of stem cell markers in differentiated cells has been re-evaluated as perhaps more meaningful than just mere dedifferentiation. It is now thought that increased cellular plasticity may have functional significance in the pathogen lifecycle and disease. In vitro cellular reprogramming of differentiated cells to pluripotency has been hailed as a way to derive pluripotent cells which has initiated the development of better research models in the lab and is under study to provide solutions in the field of regenerative medicine. Reprogramming is an epigenetic process which gradually shifts the transcriptional program of a differentiated “reprogramming” cell to that of a pluripotent one. Despite the concern for side-effects such as teratoma formation, the field of reprogramming has made progress with in vivo [35] approaches, particularly strategies to achieve tissue regeneration and rejuvenation. Studies aimed at understanding the mechanism of in vivo reprogramming, have revealed that physiological stimuli such as tissue damage, inflammation and senescence in a tissue can potentiate induced pluripotency in vivo [36]. Tissue damage or senescence triggers the release of interleukin-6 which is critical in facilitating reprogramming in neighboring cells [36]. This introduces the possibility that there may be evolutionarily conserved physiological importance for in vivo cellular reprogramming-like processes which remain to be understood. It is important to note that no findings to date suggest that such stimuli (tissue damage, senescence) can trigger pluripotency independently. However, they add to accumulating credible evidence that they may create a permissive environment for reprogramming events leading to intermediate and likely transitory stem-like states. Critically, both tissue damage and senescence are relevant to the papillomavirus lifecycle. Stem-like states do not adhere exactly to the characteristics of isolatable tissue or embryonic stem cells. However, such transitory states, are understood to form a continuum spanning between differentiated and stem cells [37,38]. They are amenable to directed differentiation in vitro, and likely represent safer avenues for transdifferentiation strategies than the use of pluripotent cells [39,40]. However, the potential roles of transitory stem-like states in physiological processes, including infection are poorly understood.

One of the most intriguing and compelling reported examples of reprogramming in vivo implicates the intracellular *Mycobacterium leprae* [41]. The bacterium has been shown to reprogram infected Schwann cells into stem cell-like cells. The reprogramming has significant implications for the course of pathogenesis as well as the life cycle of the pathogen [42]. While Schwann cells have high retention for the bacteria, the reprogrammed cells provide a route of dissemination into other cell types which may be critical for neuropathogenesis during leprosy. While the molecular pathways through which *M. leprae* infection leads to such events are incompletely understood, initial evidence suggests that they involve in part the innate immune response and inflammation which precede such reprogramming events [43].

Inflammation is naturally of relevance to the HPV lifecycle as well, particularly if one takes into account the mode of infection. Both infection-associated and sterile inflammation are understood to contribute to a regenerative response and inflammation is emerging as an evolutionarily conserved mechanism of tissue regeneration [23]. Regenerative inflammation can be mediated both via native signals to the tissue stem cells as well as to differentiated cells which may undergo profound dedifferentiation or experience increased “stemness”. In fact, in the drosophila and the mammalian gut, where regenerative inflammation has been most extensively studied, it has been shown to be important both in the maintenance of tissue homeostasis in healthy tissue, as well as the promotion of disease [23,24,26,27,44,45]. This regenerative inflammatory response is thought to extend to other epithelial tissues and is likely an important aspect of epithelial recovery following papillomavirus infection [23,46]. However, its implications in the viral lifecycle and pathogenesis have not been addressed. Newer model systems involving a murine papillomavirus which infects via a site of wounding may shed light to this aspect of papillomavirus biology as they most closely mimic the conditions of real life infection [47,48,49].

Other than leading intracellular lifestyles HPVs do not have many commonalities with mycobacteria at a first glance. However, upon closer inspection of the available evidence there is good reason to suspect that the virus may be contributing to similar events. The viral oncogenes have prominent targets which are implicated in stem cell biology and may contribute to epigenetic reprogramming via their inactivation.

## 6. Mechanisms of Enhancing Cellular Plasticity

**Figure 1:**
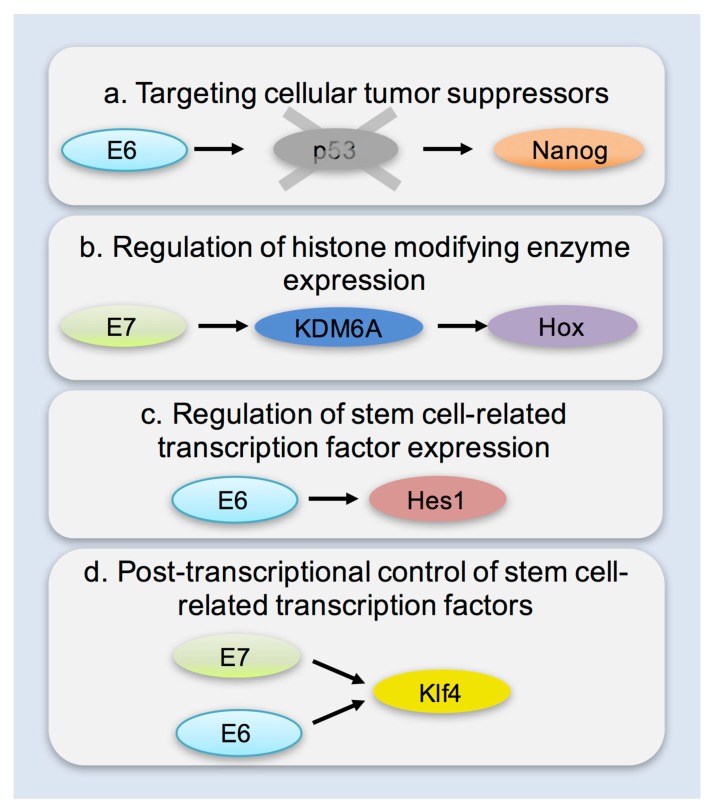
Potential mechanisms through which papillomavirus infection may contribute to the development of stem-like cells and cancer stem cells. (**a**) Targeting cellular tumor suppressors for degradation: retinoblastoma protein (pRb) has been shown to bind both sex determining region Y-box 2 (Sox2), and octamer-binding transcription factor 4 (Oct4) leading to repression of pluripotency [56]. p53 Has been shown to directly bind and suppress transcription from the Nanog promoter [55]. Both pRb and p53 have been shown to be important gatekeepers during cellular reprogramming, and their absence significantly facilitates the process [50,51,53,56]. (**b**) Transcriptional upregulation of histone modifying enzymes: Upregulation of lysine (K)-specific demethylase 6A and 6B (KDM6A and KDM6B, respectively) mediated by the *E7* oncogene of HPV16 leads to a reduction of repressive H3K27 chromatin marks and downstream activation of targets such as *Hox* genes [62,63]; (**c**) Transcriptional upregulation of stem cell-related transcription factors: the viral oncogenes *E6* and *E7* of high-risk types have been linked to the upregulation of pluripotency associated transcription factors—Oct4 [61], Hes family basic helix-loop-helix transcription factor 1 (Hes1) [67]. Infection with cutaneous papillomaviruses has also been linked to the upregulation of stem cell related genes [30,64]. (**d**) Post-transcriptional control of stem cell related transcription factors has also been demonstrated: E7 has been reported to bind Oct4 and act as a transcriptional co-activator [60]. Both E6 and E7 have been shown to transcriptionally, post-transcriptionally and post-translationally regulate Kruppel-like factor 4 (Klf4) (e.g., via hyposumoylation) leading to modified Klf4 activity in infected keratinocytes [58]. The upregulation of stemness related genes has been most frequently attributed to the viral oncogenes *E6* and *E7* but the full mechanisms underlying some of these effects have yet to be elucidated. Furthermore, the impact of the re-expression or modulation of stemness related genes in the viral lifecycle and carcinogenesis is still poorly understood.

Work aimed at illuminating the critical steps during the process of reprogramming cells to pluripotency, clearly implicated prominent tumor suppressors in stem cell biology. p53, retinoblastoma protein (pRb) and other key players in tumor suppression have been shown to control checkpoints during cellular reprogramming [50,51,52,53,54]. Their loss or inhibition has been shown to facilitate the reprogramming process. Furthermore, they have been shown to impact the function of stem cell related factors: p53 can control the expression of the stem cell related protein Nanog [55], while pRb can directly bind sex determining region Y-box 2 (Sox2),and octamer-binding transcription factor 4 (Oct4) [56] thus controlling pluripotency networks via these key transcription factors. Since both p53 and pRb are critical targets of papillomavirus oncogenes it is important to interrogate whether in addition to removing critical barriers for cell cycle control, their targeting during viral infection creates a tissue environment which is more conducive to increased cellular plasticity (Figure 1). Consistent with this E6 contributed to reprogramming of cells from Fanconi anemia patients which are difficult to reprogram via its action on p53 [57]. The proteins encoded by the *INK4A/ARF* locus, some of which are implicated in papillomavirus pathogenesis are also important during reprogramming [52]. The extent to which in vivo inactivation of such tumor suppressors enables increased cellular plasticity and phenomena of natural reprogramming remains to be seen.

In addition to targeting tumor suppressors which have been linked to cellular reprogramming there is also evidence to suggest that the viral oncogenes may contribute to cellular reprogramming in ways which are independent of their ability to target p53 or pRb. High-risk HPVs (16, 31) have recently been shown to upregulate Kruppel-like factor 4 (Klf4) and contribute to its hypoSUMOylation [58]. This upregulation was necessary for the differentiation dependent lifecycle of the virus however it is also critical to note that the functions of Klf4 cells harboring viral genomes were markedly different to those seen in control keratinocytes. Biochemical evidence which dates back to the initial understanding of the function of Oct4 demonstrated the ability of high-risk HPV E7 (similar oncoproteins such as adenovirus E1A) to directly interact and synergize with Oct4 for the activation of its target genes [59,60]. More recent evidence from transgenic animals suggests that E7 may also contribute to the transcriptional upregulation of Oct4 [61]. Furthermore, E7 was shown to epigenetically reprogram cells via the transcriptional activation of histone demethylases lysine (K)-specific demethylase 6A and 6B (KDM6A and KDM6B, respectively) [62]. This upregulation led to a decrease in Histone3 Lysine27 (H3K27) trimethylation, and downstream transcriptional changes such as the activation of *Hox* genes. Critically this expression has been linked to the high p16 expression characteristic of HPV positive cancers and may represent a way of targeting HPV-positive cancer cells since they are dependent on its continued expression [63]. The E7 protein of cutaneous HPVs has been shown to lead to the upregulation of several stem cell markers genes in infected keratinocytes and to the formation of cells with stem-like properties—e.g., epithelial cell adhesion molecule (EpCaM) [64]. High-risk mucosal as well as cutaneous HPVs and murine papillomaviruses also inhibit the Notch pathway which is known to play important roles in commitment to differentiation [65,66]. Of course, the role of such reprogramming to the viral lifecycle and pathogenesis is not well understood. It may be linked to important aspects of the viral life cycle.

## 7. Potential Links of Cellular Plasticity to Disease

There is a dearth of studies which would allow definitive conclusions about the upregulation of stem cell related proteins in the viral life cycle. However, there is an accumulating body of evidence suggesting they may be linked to disease. The upregulation of genes related to stem cells may be a way in which the virus contributes to the formation and maintenance of cancer stem cell populations (Figure 1). Recently E6 has been shown to be upregulated in cancer stem cell-like cells isolated from primary tumors cervical cell lines based on their immunophenotype and sphere forming capabilities [67]. These cells were also shown to express Oct4, Sox2, Nanog, and Lrig1 and were dependent on E6-mediated expression of Hes1 for continued self-renewal.

The expression of stem cell related markers is now increasingly reported in HPV-related cancers. Proteins such as Sox2, Nanog, and Oct4 are now thought to serve not only as biomarkers tumor stage and therapy response in cancer but also may be directly implicated in the process of carcinogenesis. In HPV-associated cancers stem cell markers Oct4, Sox2 (in cervical cancers) [68], and Lrig1 (in head and neck squamous cell carcinomas) [69] have been reported and proposed as potential biomarkers. Given the established role of p16^INK4A^ as a useful biomarker it remains to be seen whether the use of these are of added prognostic benefit. Nevertheless, the expression of these proteins in cancer biopsies and cervical cancer cell lines may provide insights into potential roles in the carcinogenic process. Critically, the links of early gene products, particularly the viral oncogenes of human papillomaviruses to the upregulation of such markers lends credibility to this notion.

A source of skepticism for studies supporting a changing cellular state during infection, derives from the refractoriness of keratinocytes to cellular reprogramming and perceived lack of plasticity. It is clear that no condition has been described thus far where a viral gene product or set of products have been shown to independently lead to an isolatable stem cell. However, it is critical to bear in mind that infection occurs in the context of a wound. It is an aspect of infection which has been poorly accounted for particularly since many experimental models used in papillomavirus research do not take it into consideration. In virion-infected monolayer keratinocytes, organotypic cultures of stably transfected keratinocytes or transgenic animals expressing viral gene products, the wound environment is not routinely modelled. However, during real-life infection, in addition to the viral invasion which triggers an inflammatory response via the presence of pathogen associated molecular patterns (PAMPs) there are also damage associated molecular patterns (DAMPs), and reactive oxygen species (ROS) released as a result of the wounding. Thus, both infection-associated, as well as sterile inflammation should be kept in mind as additional important factors which likely remove constraints towards increased cellular plasticity [36].

## 8. Conclusions

**Figure 2:**
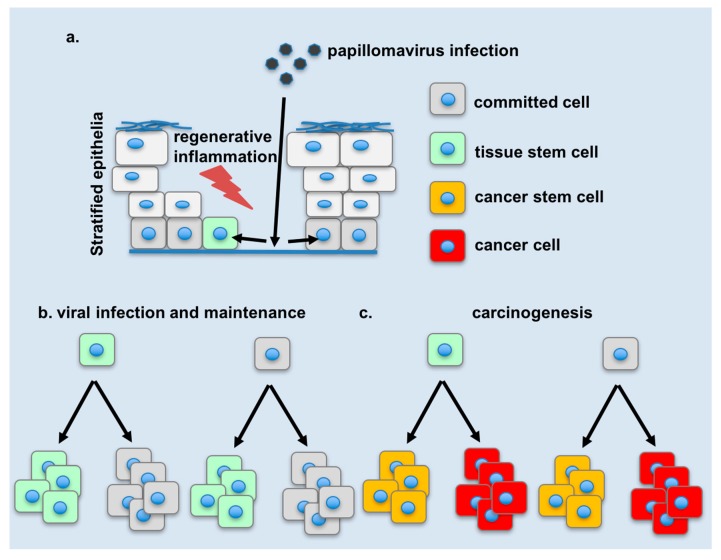
Model of changes in stem cells during papillomavirus infection and carcinogenesis. (**a**) Papillomaviruses can gain access to the basal layer of stratified epithelia via microwounds. Following attachment to the basement membrane, the virus can infect committed or stem cells in the basal layer of the epithelium. As the epithelium heals, infected cells are subject to changes due to the expression of early gene products and local inflammatory signals linked to infection and the regenerative response. (**b**) Stem cells or stem-like cells (with at least some atypical features) detected during infection and viral maintenance may be derived from infected stem cells or committed cells which have been reprogrammed. (**c**) Cancer stem cells may likewise be derived from infected tissue stem cells or from drastically de-differentiated committed cells.

The role of stem cells and cellular stemness has been proposed to be important in the lifecycle and disease promotion by HPVs. Viral genomes have been detected in anatomical locations which are consistent with those of stem cells yet conclusive evidence as to whether infected stem cells are the sites of viral maintenance of all HPVs or the cancer initiating cells of HPV-related cancers is to this day tenuous. Early gene products, and particularly the viral oncogenes have been shown to modify stem cell dynamics and cellular stemness, however the extent to which this is critical for the viral lifecycle or for ensuing disease remains elusive. As HPV-related cancers account for about 5% of human cancers [13] (and potentially more if one considers the accumulating evidence for the role of β-papillomaviruses in cutaneous carcinogenesis) understanding the viral reservoir and mechanisms of carcinogenesis remains imperative. At the same time, emerging tools and concepts from the booming field of stem cell biology facilitate the task at hand. Current evidence suggests that viral maintenance occurs in cells whose characteristics are consistent to those of stem cells (Figure 2). Critical questions remain in tracing the origins of such cells: do they derive from infected tissue stem cells, or do they develop stem-like characteristics subsequent to infection? It is also clear that early gene products, and particularly the viral oncogenes can change the behavior of stem cells in a tissue, as well as reprogram cells towards states which resemble stem cells in some aspects. The next frontier would be to uncover direct links for these phenomena to aspects of the lifecycle on carcinogenesis.

## References

[1] Kines R.C., Thompson C.D., Lowy D.R., Schiller J.T., Day P.M. (2009). The initial steps leading to papillomavirus infection occur on the basement membrane prior to cell surface binding. Proc. Natl. Acad. Sci. USA.

[2] Schiller J.T., Day P.M., Kines R.C. (2010). Current understanding of the mechanism of HPV infection. Gynecol. Oncol..

[3] Mascre G., Dekoninck S., Drogat B., Youssef K.K., Brohee S., Sotiropoulou P.A., Simons B.D., Blanpain C. (2012). Distinct contribution of stem and progenitor cells to epidermal maintenance. Nature.

[4] Doorbar J. (2013). Latent papillomavirus infections and their regulation. Curr. Opin. Virol..

[5] Maglennon G.A., McIntosh P., Doorbar J. (2011). Persistence of viral DNA in the epithelial basal layer suggests a model for papillomavirus latency following immune regression. Virology.

[6] Da Silva-Diz V., Sole-Sanchez S., Valdes-Gutierrez A., Urpi M., Riba-Artes D., Penin R.M., Pascual G., Gonzalez-Suarez E., Casanovas O., Vinals F. (2013). Progeny of Lgr5-expressing hair follicle stem cell contributes to papillomavirus-induced tumor development in epidermis. Oncogene.

[7] Kranjec C., Doorbar J. (2016). Human papillomavirus infection and induction of neoplasia: A matter of fitness. Curr. Opin. Virol..

[8] Fehrmann F., Laimins L.A. (2003). Human papillomaviruses: Targeting differentiating epithelial cells for malignant transformation. Oncogene.

[9] Hong S., Laimins L.A. (2013). Regulation of the life cycle of hpvs by differentiation and the DNA damage response. Future Microbiol..

[10] Ito M., Liu Y., Yang Z., Nguyen J., Liang F., Morris R.J., Cotsarelis G. (2005). Stem cells in the hair follicle bulge contribute to wound repair but not to homeostasis of the epidermis. Nat. Med..

[11] Blanpain C., Fuchs E. (2014). Stem cell plasticity. Plasticity of epithelial stem cells in tissue regeneration. Science.

[12] Rompolas P., Mesa K.R., Greco V. (2013). Spatial organization within a niche as a determinant of stem-cell fate. Nature.

[13] (2007). Human papillomaviruses. IARC Monographs on the Evaluation of Carcinogenic Risks to Humans.

[14] Brown D.R., Weaver B. (2013). Human papillomavirus in older women: New infection or reactivation?. J. Infect. Dis..

[15] Gravitt P.E., Rositch A.F., Silver M.I., Marks M.A., Chang K., Burke A.E., Viscidi R.P. (2013). A cohort effect of the sexual revolution may be masking an increase in human papillomavirus detection at menopause in the united states. J. Infect. Dis..

[16] Schmitt A., Rochat A., Zeltner R., Borenstein L., Barrandon Y., Wettstein F.O., Iftner T. (1996). The primary target cells of the high-risk cottontail rabbit papillomavirus colocalize with hair follicle stem cells. J. Virol..

[17] Boxman I.L., Berkhout R.J., Mulder L.H., Wolkers M.C., Bouwes Bavinck J.N., Vermeer B.J., Ter Schegget J. (1997). Detection of human papillomavirus dna in plucked hairs from renal transplant recipients and healthy volunteers. J. Investig. Dermatol..

[18] De Koning M.N., Struijk L., Bavinck J.N., Kleter B., Ter Schegget J., Quint W.G., Feltkamp M.C. (2007). β-papillomaviruses frequently persist in the skin of healthy individuals. J. Gen. Virol..

[19] Galloway D.A., Laimins L.A. (2015). Human papillomaviruses: Shared and distinct pathways for pathogenesis. Curr. Opin. Virol..

[20] Tommasino M. (2017). The biology of β-human papillomaviruses. Virus Res..

[21] Martens J.E., Arends J., Van der Linden P.J., De Boer B.A., Helmerhorst T.J. (2004). Cytokeratin 17 and p63 are markers of the HPV target cell, the cervical stem cell. Anticancer Res..

[22] Herfs M., Yamamoto Y., Laury A., Wang X., Nucci M.R., McLaughlin-Drubin M.E., Munger K., Feldman S., McKeon F.D., Xian W. (2012). A discrete population of squamocolumnar junction cells implicated in the pathogenesis of cervical cancer. Proc. Natl. Acad. Sci. USA.

[23] Karin M., Clevers H. (2016). Reparative inflammation takes charge of tissue regeneration. Nature.

[24] Nigro G., Rossi R., Commere P.H., Jay P., Sansonetti P.J. (2014). The cytosolic bacterial peptidoglycan sensor Nod2 affords stem cell protection and links microbes to gut epithelial regeneration. Cell Host Microbe.

[25] Neal M.D., Sodhi C.P., Jia H., Dyer M., Egan C.E., Yazji I., Good M., Afrazi A., Marino R., Slagle D. (2012). Toll-like receptor 4 is expressed on intestinal stem cells and regulates their proliferation and apoptosis via the p53 up-regulated modulator of apoptosis. J. Biol. Chem..

[26] Apidianakis Y., Pitsouli C., Perrimon N., Rahme L. (2009). Synergy between bacterial infection and genetic predisposition in intestinal dysplasia. Proc. Natl. Acad. Sci. USA.

[27] Pitsouli C., Apidianakis Y., Perrimon N. (2009). Homeostasis in infected epithelia: Stem cells take the lead. Cell Host Microbe.

[28] Mysorekar I.U., Isaacson-Schmid M., Walker J.N., Mills J.C., Hultgren S.J. (2009). Bone morphogenetic protein 4 signaling regulates epithelial renewal in the urinary tract in response to uropathogenic infection. Cell Host Microbe.

[29] Michael S., Lambert P.F., Strati K. (2013). The HPV16 oncogenes cause aberrant stem cell mobilization. Virology.

[30] Lanfredini S., Olivero C., Borgogna C., Calati F., Powell K., Davies K.J., De Andrea M., Harries S., Tang H.K.C., Pfister H. (2017). HPV8 field cancerization in a transgenic mouse model is due to Lrig1+ keratinocyte stem cell expansion. J. Investig. Dermatol..

[31] White A.C., Khuu J.K., Dang C.Y., Hu J., Tran K.V., Liu A., Gomez S., Zhang Z., Yi R., Scumpia P. (2014). Stem cell quiescence acts as a tumour suppressor in squamous tumours. Nat. Cell. Biol..

[32] Jaks V., Barker N., Kasper M., Van Es J.H., Snippert H.J., Clevers H., Toftgård R. (2008). Lgr5 marks cycling, yet long-lived, hair follicle stem cells. Nat. Genet..

[33] Lin K.K., Andersen B. (2008). Have hair follicle stem cells shed their tranquil image?. Cell Stem Cell.

[34] Takahashi K., Yamanaka S. (2006). Induction of pluripotent stem cells from mouse embryonic and adult fibroblast cultures by defined factors. Cell.

[35] Abad M., Mosteiro L., Pantoja C., Canamero M., Rayon T., Ors I., Grana O., Megias D., Dominguez O., Martinez D. (2013). Reprogramming in vivo produces teratomas and iPS cells with totipotency features. Nature.

[36] Mosteiro L., Pantoja C., Alcazar N., Marión R.M., Chondronasiou D., Rovira M., Fernandez-Marcos P.J., Muñoz-Martin M., Blanco-Aparicio C., Pastor J. (2016). Tissue damage and senescence provide critical signals for cellular reprogramming in vivo. Science.

[37] Stadtfeld M., Maherali N., Breault D.T., Hochedlinger K. (2008). Defining molecular cornerstones during fibroblast to iPS cell reprogramming in mouse. Cell Stem Cell.

[38] Hochedlinger K., Plath K. (2009). Epigenetic reprogramming and induced pluripotency. Development.

[39] Kelaini S., Cochrane A., Margariti A. (2014). Direct reprogramming of adult cells: Avoiding the pluripotent state. Stem Cells Cloning.

[40] Margariti A., Winkler B., Karamariti E., Zampetaki A., Tsai T.N., Baban D., Ragoussis J., Huang Y., Han J.D., Zeng L. (2012). Direct reprogramming of fibroblasts into endothelial cells capable of angiogenesis and reendothelialization in tissue-engineered vessels. Proc. Natl. Acad. Sci. USA.

[41] Masaki T., Qu J., Cholewa-Waclaw J., Burr K., Raaum R., Rambukkana A. (2013). Reprogramming adult schwann cells to stem cell-like cells by leprosy bacilli promotes dissemination of infection. Cell.

[42] Masaki T., McGlinchey A., Tomlinson S.R., Qu J., Rambukkana A. (2013). Reprogramming diminishes retention of mycobacterium leprae in schwann cells and elevates bacterial transfer property to fibroblasts. F1000Research.

[43] Masaki T., McGlinchey A., Cholewa-Waclaw J., Qu J., Tomlinson S.R., Rambukkana A. (2014). Innate immune response precedes *Mycobacterium leprae*-induced reprogramming of adult Schwann cells. Cell. Reprogram..

[44] Panayidou S., Apidianakis Y. (2013). Regenerative inflammation: Lessons from *Drosophila* intestinal epithelium in health and disease. Pathogens.

[45] Taniguchi K., Wu L.W., Grivennikov S.I., De Jong P.R., Lian I., Yu F.X., Wang K., Ho S.B., Boland B.S., Chang J.T. (2015). A gp130-Src-YAP module links inflammation to epithelial regeneration. Nature.

[46] Michael S., Achilleos C., Panayiotou T., Strati K. (2016). Inflammation shapes stem cells and stemness during infection and beyond. Front. Cell Dev. Biol..

[47] Ingle A., Ghim S., Joh J., Chepkoech I., Bennett Jenson A., Sundberg J.P. (2011). Novel laboratory mouse papillomavirus (MusPV) infection. Vet. Pathol..

[48] Handisurya A., Day P.M., Thompson C.D., Buck C.B., Pang Y.Y., Lowy D.R., Schiller J.T. (2013). Characterization of *Mus musculus* papillomavirus 1 infection in situ reveals an unusual pattern of late gene expression and capsid protein localization. J. Virol..

[49] Uberoi A., Yoshida S., Frazer I.H., Pitot H.C., Lambert P.F. (2016). Role of ultraviolet radiation in papillomavirus-induced disease. PLoS Pathog..

[50] Kawamura T., Suzuki J., Wang Y.V., Menendez S., Morera L.B., Raya A., Wahl G.M., Izpisua Belmonte J.C. (2009). Linking the p53 tumour suppressor pathway to somatic cell reprogramming. Nature.

[51] Marion R.M., Strati K., Li H., Murga M., Blanco R., Ortega S., Fernandez-Capetillo O., Serrano M., Blasco M.A. (2009). A p53-mediated DNA damage response limits reprogramming to ensure iPS cell genomic integrity. Nature.

[52] Li H., Collado M., Villasante A., Strati K., Ortega S., Canamero M., Blasco M.A., Serrano M. (2009). The Ink4/Arf locus is a barrier for ips cell reprogramming. Nature.

[53] Marion R.M., Strati K., Li H., Tejera A., Schoeftner S., Ortega S., Serrano M., Blasco M.A. (2009). Telomeres acquire embryonic stem cell characteristics in induced pluripotent stem cells. Cell Stem Cell.

[54] Utikal J., Polo J.M., Stadtfeld M., Maherali N., Kulalert W., Walsh R.M., Khalil A., Rheinwald J.G., Hochedlinger K. (2009). Immortalization eliminates a roadblock during cellular reprogramming into iPS cells. Nature.

[55] Lin T., Chao C., Saito S., Mazur S.J., Murphy M.E., Appella E., Xu Y. (2005). P53 induces differentiation of mouse embryonic stem cells by suppressing nanog expression. Nat. Cell. Biol..

[56] Kareta M.S., Gorges L.L., Hafeez S., Benayoun B.A., Marro S., Zmoos A.F., Cecchini M.J., Spacek D., Batista L.F., O’Brien M. (2015). Inhibition of pluripotency networks by the Rb tumor suppressor restricts reprogramming and tumorigenesis. Cell Stem Cell.

[57] Chlon T.M., Hoskins E.E., Mayhew C.N., Wikenheiser-Brokamp K.A., Davies S.M., Mehta P., Myers K.C., Wells J.M., Wells S.I. (2014). High-risk human papillomavirus E6 protein promotes reprogramming of fanconi anemia patient cells through repression of p53 but does not allow for sustained growth of induced pluripotent stem cells. J. Virol..

[58] Gunasekharan V.K., Li Y., Andrade J., Laimins L.A. (2016). Post-transcriptional regulation of Klf4 by high-risk human papillomaviruses is necessary for the differentiation-dependent viral life cycle. PLoS Pathog..

[59] Brehm A., Ohbo K., Scholer H. (1997). The carboxy-terminal transactivation domain of Oct-4 acquires cell specificity through the POU domain. Mol. Cell. Biol..

[60] Brehm A., Ohbo K., Zwerschke W., Botquin V., Jansen-Durr P., Scholer H.R. (1999). Synergism with germ line transcription factor Oct-4: Viral oncoproteins share the ability to mimic a stem cell-specific activity. Mol. Cell. Biol..

[61] Organista-Nava J., Gómez-Gómez Y., Ocadiz-Delgado R., García-Villa E., Bonilla-Delgado J., Lagunas-Martínez A., Tapia J.S., Lambert P.F., García-Carrancá A., Gariglio P. (2016). The HPV16 E7 oncoprotein increases the expression of Oct3/4 and stemness-related genes and augments cell self-renewal. Virology.

[62] McLaughlin-Drubin M.E., Crum C.P., Munger K. (2011). Human papillomavirus E7 oncoprotein induces KDM6A and KDM6B histone demethylase expression and causes epigenetic reprogramming. Proc. Natl. Acad. Sci. USA.

[63] McLaughlin-Drubin M.E., Park D., Munger K. (2013). Tumor suppressor p16^INK4A^ is necessary for survival of cervical carcinoma cell lines. Proc. Natl. Acad. Sci. USA.

[64] Hufbauer M., Biddle A., Borgogna C., Gariglio M., Doorbar J., Storey A., Pfister H., Mackenzie I., Akgül B. (2013). Expression of β-papillomavirus oncogenes increases the number of keratinocytes with stem cell-like properties. J. Virol..

[65] Meyers J.M., Uberoi A., Grace M., Lambert P.F., Munger K. (2017). Cutaneous HPV8 and MmuPV1 E6 proteins target the Notch and TGF-β tumor suppressors to inhibit differentiation and sustain keratinocyte proliferation. PLoS Pathog..

[66] Kranjec C., Holleywood C., Libert D., Griffin H., Mahmood R., Isaacson E., Doorbar J. (2017). Modulation of basal cell fate during productive and transforming HPV16 infection is mediated by progressive E6-driven depletion of Notch. J. Pathol..

[67] Tyagi A., Vishnoi K., Mahata S., Verma G., Srivastava Y., Masaldan S., Roy B.G., Bharti A.C., Das B.C. (2016). Cervical cancer stem cells selectively overexpress hpv oncoprotein E6 that controls stemness and self-renewal through upregulation of Hes1. Clin. Cancer Res..

[68] Kim B.W., Cho H., Choi C.H., Ylaya K., Chung J.Y., Kim J.H., Hewitt S.M. (2015). Clinical significance of Oct4 and Sox2 protein expression in cervical cancer. BMC Cancer.

[69] Lindquist D., Näsman A., Tarján M., Henriksson R., Tot T., Dalianis T., Hedman H. (2014). Expression of LRIG1 is associated with good prognosis and human papillomavirus status in oropharyngeal cancer. Br. J. Cancer.

